# Subject-Specific Modeling of Femoral Torsion Influences the Prediction of Hip Loading During Gait in Asymptomatic Adults

**DOI:** 10.3389/fbioe.2021.679360

**Published:** 2021-07-21

**Authors:** Enrico De Pieri, Bernd Friesenbichler, Renate List, Samara Monn, Nicola C. Casartelli, Michael Leunig, Stephen J. Ferguson

**Affiliations:** ^1^Laboratory for Movement Analysis, University of Basel Children’s Hospital, Basel, Switzerland; ^2^Department of Biomedical Engineering, University of Basel, Basel, Switzerland; ^3^Institute for Biomechanics, ETH Zurich, Zürich, Switzerland; ^4^Human Performance Lab, Schulthess Clinic, Zürich, Switzerland; ^5^Laboratory of Exercise and Health, ETH Zurich, Schwerzenbach, Switzerland; ^6^Department of Orthopaedic Surgery, Schulthess Clinic, Zürich, Switzerland

**Keywords:** femoral torsion, hip osteoarthritis, hip contact forces, muscle lever arms, musculoskeletal modeling, EOS imaging

## Abstract

Hip osteoarthritis may be caused by increased or abnormal intra-articular forces, which are known to be related to structural articular cartilage damage. Femoral torsional deformities have previously been correlated with hip pain and labral damage, and they may contribute to the onset of hip osteoarthritis by exacerbating the effects of existing pathoanatomies, such as cam and pincer morphologies. A comprehensive understanding of the influence of femoral morphotypes on hip joint loading requires subject-specific morphometric and biomechanical data on the movement characteristics of individuals exhibiting varying degrees of femoral torsion. The aim of this study was to evaluate hip kinematics and kinetics as well as muscle and joint loads during gait in a group of adult subjects presenting a heterogeneous range of femoral torsion by means of personalized musculoskeletal models. Thirty-seven healthy volunteers underwent a 3D gait analysis at a self-selected walking speed. Femoral torsion was evaluated with low-dosage biplanar radiography. The collected motion capture data were used as input for an inverse dynamics analysis. Personalized musculoskeletal models were created by including femoral geometries that matched each subject’s radiographically measured femoral torsion. Correlations between femoral torsion and hip kinematics and kinetics, hip contact forces (HCFs), and muscle forces were analyzed. Within the investigated cohort, higher femoral antetorsion led to significantly higher anteromedial HCFs during gait (medial during loaded stance phase and anterior during swing phase). Most of the loads during gait are transmitted through the anterior/superolateral quadrant of the acetabulum. Correlations with hip kinematics and muscle forces were also observed. Femoral antetorsion, through altered kinematic strategies and different muscle activations and forces, may therefore lead to altered joint mechanics and pose a risk for articular damage. The method proposed in this study, which accounts for both morphological and kinematic characteristics, might help in identifying in a clinical setting patients who, as a consequence of altered femoral torsional alignment, present more severe functional impairments and altered joint mechanics and are therefore at a higher risk for cartilage damage and early onset of hip osteoarthritis.

## Introduction

Increased or abnormal intra-articular forces can lead to structural damages to the articular cartilage, loss of joint integrity, and tissue degeneration and thus to hip osteoarthritis (OA) (Solomon, 1976; Klaue et al., 1991; Tanzer and Noiseux, 2004; Ganz et al., 2008; Felson, 2013). The altered stresses in localized areas of the cartilage are often determined by a combination of overall excessive loading, due for instance to obesity or intense physical activities, as well as by morphological abnormalities in the hip joint structures (Felson, 2013).

Two main types of hip morphology have been identified as potential risk factors for developing hip OA: Hip dysplasia and morphologies associated with femoroacetabular impingement (FAI) syndrome, that is, cam morphology (reduced femoral head–neck offset) or pincer morphology (deep and/or retroverted acetabulum) (Thomas et al., 2012; Felson, 2013). However, other morphological features, such as femoral torsion, may contribute to the onset of hip OA, as it may exacerbate or diminish the effects of an existing cam and/or pincer morphology (Zeng et al., 2016). Indeed, femoral torsion can considerably affect impingement-free hip range of motion, aggravating or compensating excessive cartilage loading caused by concomitant cam/pincer deformities (Schmaranzer et al., 2019).

Femoral torsional and coronal deformities have previously been correlated with hip pain and labral damage (Tönnis and Heinecke, 1999). The presence of structural hip abnormalities is often observed in patients presenting labral tears (Wenger et al., 2004). Another study documented that among patients who underwent hip arthroscopy for labral pathology or FAI, the ones with higher femoral antetorsion presented larger and more anterior labral tears (Ejnisman et al., 2013). Femoral torsion is known to have a strong influence on the loading environment in the proximal femur and the hip (Heller et al., 2001). Increased femoral torsion has also been associated with complications in the adjacent joints, such as knee pain and OA (Eckhoff et al., 1997; Bretin et al., 2011), patellar instability, and pain (Powers, 2003; Stevens et al., 2014). Furthermore, abnormal values of femoral torsion could also represent a risk factor for hip dislocation (Upadhyay et al., 1985; Novais et al., 2019).

Femoral deformities that have been linked to hip joint degeneration are understood to represent developmental variations of normal human anatomy (Hogervorst et al., 2011, 2012). In particular, the amount of femoral torsion depends on age and sex (Hetsroni et al., 2013), starting approximatively at 40° at birth and ranging between 15° and 20° during adulthood (Fabry et al., 1973). Developmental torsional deformities of the lower limb in children and adolescents represent a frequent reason for consultation with pediatric orthopedic clinicians (Fabry, 2010). In addition to cosmetic considerations regarding their gait (Fabry, 2010), these deformities have been associated with the development of gait impairment and joint pain (Bruderer-Hofstetter et al., 2015). Nevertheless, there is no consistent definition of what can be considered excessive or pathological femoral antetorsion, with threshold values spanning between > 30° and > 50° (Jani et al., 1979; Cordier and Katthagen, 2000; Hefti, 2000; Alexander et al., 2019). Moreover, optimal surgical treatment for mechanically induced hip pain depends upon understanding the forces that are produced within the acetabulum and the potential mechanical consequences of femoral reorientation.

There is a general understanding that the rotational alignment of the whole lower limb plays a critical role in the onset of hip pathologies (Eckhoff, 1994; Keshmiri et al., 2016). Additionally, several kinematic compensatory strategies can be adopted at the hip (Bruderer-Hofstetter et al., 2015; Alexander et al., 2019), as a consequence of either pain avoidance (Leigh et al., 2016; Popovic et al., 2020) or lever arm dysfunction, particularly of the hip abductors (Arnold et al., 1997; van der Krogt et al., 2012; Boyer et al., 2016). Moreover, modeling excessive femoral antetorsion was shown to alter magnitude and orientation of predicted joint contact forces in pediatric pathological populations (Bosmans et al., 2014; Passmore et al., 2018), as well as in typically developing children (Kainz et al., 2020).

A complete understanding of the influence of femoral antetorsion on hip joint loading requires subject-specific morphometric and biomechanical data on the movement characteristics of individuals exhibiting varying degrees of femoral torsion. Musculoskeletal modeling allows us to estimate muscle activations and intra-articular joint forces (Erdemir et al., 2007) in relation to individual motion patterns and musculoskeletal geometry and represents therefore a suitable tool for a comprehensive evaluation of the lower-limb biomechanics in association with femoral torsional morphologies. Musculoskeletal models are commonly built starting from a cadaveric-based model template, which is scaled or morphed to match the overall anthropometrics of an individual subject (Andersen, 2021). However, these scaled models may not necessarily resemble the real anatomy of a subject, particularly when his/her bone morphology largely deviates from the baseline cadaveric template. The inclusion of additional subject-specific parameters, such as femoral torsion, may reduce the uncertainty associated with model scaling.

The aim of this study was to evaluate hip kinematics and kinetics, hip contact forces (HCFs), and muscle forces during gait in a group of asymptomatic adult subjects presenting a heterogeneous range of femoral torsion. It was hypothesized that increased femoral torsion may lead to alterations in hip kinematics and loading. Specifically, higher femoral torsion was expected to alter muscle lever arms and potentially lead to kinematic compensations, thus influencing the muscle recruitment pattern and the predicted required muscle forces and therefore determining changes in the resulting HCFs. In order to achieve this, personalized musculoskeletal models were created based on three-dimensional (3D) gait analysis data and morphological data extracted from low-dosage biplanar radiographic imaging. The effect of modeling subject-specific femoral torsion was additionally investigated by qualitatively analyzing changes in muscle lever arms for a broad range of hip motions and by comparing, in the examined cohort, HCFs predicted with personalized and generic models during gait.

## Materials and Methods

### Participants

Thirty-seven healthy volunteers (27.7 ± 4.6 years old, 15 females, mean BMI = 23.0 ± 2.6) were recruited for this study. Subjects between 18 and 50 years of age were considered eligible for the study if they did not present back or lower-extremity pain at the time of the study; any surgery or significant injury on the back or lower extremities in the last 12 months; a history of open or arthroscopic hip surgery; known conditions affecting gait, balance, or physical activities; a BMI over 35 kg/m^2^; or pregnancy.

### Radiographic Data

A full-length radiograph of the lower limbs was acquired for each subject using a low-dosage biplanar EOS system (EOS Imaging Inc., France) (Folinais et al., 2013). An effective radiation dose lower than 0.63 mSv guaranteed minimal risks to the participants (Mettler et al., 2008; Buck et al., 2012; Rosskopf et al., 2016). Femoral torsion was assessed on 3D reconstructions of the femur, utilizing the biplanar EOS radiographs (sterEOS software, EOS Imaging Inc., France) (Buck et al., 2012). It was calculated as the angle between a line through the femoral neck and a line adapted to the posterior contour of the femoral condyles (Hernandez et al., 1981). The line through the femoral neck was defined as the midline between the cortices of the femoral neck through the caudally projected center of the femoral head. Antetorsion was defined as the clockwise rotation of the proximal relative to the distal femur.

Data from a randomly chosen leg for each subject were included in the analysis. Measured torsional values ranged from –7° of retrotorsion to + 38° of antetorsion, with a mean value of 16.2° ± 10.0°. The average difference between the two limbs of each subject was 4.2° (range: 0.1°–14.5°).

### Motion-Capture Data

Lower-limb kinematics and kinetics were collected during gait using a 13-camera motion-capture system (Vicon Motion Systems Ltd., Oxford, United Kingdom) capturing at 200 Hz, synchronized with three force plates (Kistler Instrumentation, Winterthur, Switzerland) sampling at 1,000 Hz.

The lower extremities were equipped with 47 skin-mounted markers according to the IfB markers set (List et al., 2013) and extended with three markers on the trunk.

After the acquisition of a standing trial in an anatomic upright position, each subject completed five successful stride cycles for each leg. Walking speed was self-selected but controlled to be within ± 10% of the first assessed trial (cohort mean = 5.21 km/h, SD = 0.58 km/h).

All markers were labeled and gap-filled in Vicon Nexus (versions 2.8, 2.9, 2.10, Vicon, Oxford, United Kingdom). Kinematic data were filtered using a low-pass (10 Hz, fourth order) Butterworth filter. Ground reaction force (GRF) data were filtered using a low-pass Butterworth filter (20 Hz, fourth order), and heel strike and toe-off were determined from the GRF measurements using force thresholds (> 20 N for heel strike and < 20 N for toe-off).

### Musculoskeletal Modeling

Musculoskeletal modeling was performed with a commercially available software (AnyBody Modeling System, version 7.3, Aalborg, Denmark) (Damsgaard et al., 2006), using motion-capture data as input.

**FIGURE 1:**
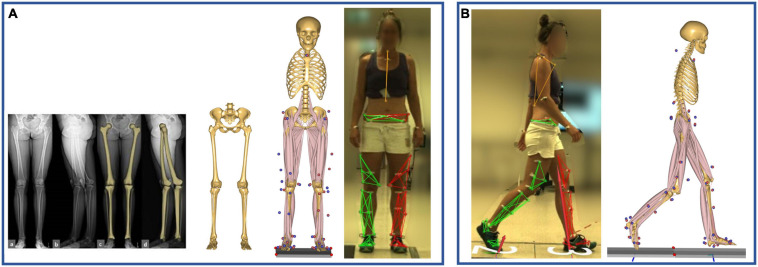
**(A)** Model scaling and personalization based on subject-specific radiographic measurements, obtained through a low-dosage biplanar EOS system (EOS Imaging Inc., France). The distance between the hip joint centers, measured in the frontal plane, was used to scale the pelvic width, while the femoral torsion was calculated through 3D reconstructions of the femurs (sterEOS software, EOS Imaging Inc., France). The model was scaled to match the subject’s anthropometrics based on marker data collected during a standing reference trial. **(B)** Kinematic and kinetic analyses during gait were based on the tracking of the measured marker trajectories.

Personalized models were created from a detailed musculoskeletal model of the lower limb (De Pieri et al., 2018), based on a cadaveric dataset (Carbone et al., 2015), which was scaled to match the anthropometrics of each patient and the marker data collected during a standing reference trial (Lund et al., 2015; Figure 1A). The hip joint is modeled as a 3-degrees of freedom (DOF) ball-and-socket joint, while knee, talocrural, and subtalar joints are modeled as 1-DOF hinges. Additionally, the position of the patella is defined as a function of the knee flexion angle. The distance between hip joint centers (HJCs) measured in the radiographic images was used as a reference for scaling the pelvic width to improve the models’ accuracy (Fischer et al., 2018). In each subject-specific model, both femurs were morphed to include a transversal rotation between the proximal and distal sections, matching the subject’s femoral torsion measured from the radiographic data (Figure 1A). The femoral morphing was based on radial basis function 3D transformation.

A kinematic analysis based on the marker trajectories was conducted to compute joint kinematics (Andersen et al., 2009; Lund et al., 2015; Figure 1B). Secondly, an inverse dynamics analysis, based on a third-order-polynomial muscle recruitment criterion, was performed to calculate required muscle activations and forces, as well as resulting joint moments and contact forces (Andersen, 2021).

As the spatial orientation of the femoral segment in the global reference frame is determined by the positions of the bony-landmark-based markers, the inclusion of a morphed femoral geometry did not affect the calculation of joint kinematics, rather just the position of muscles’ origin and insertion points along the femur and their lines of action relative to the adjacent joints.

### Femoral Torsion and Muscle Lever Arms

**FIGURE 2:**
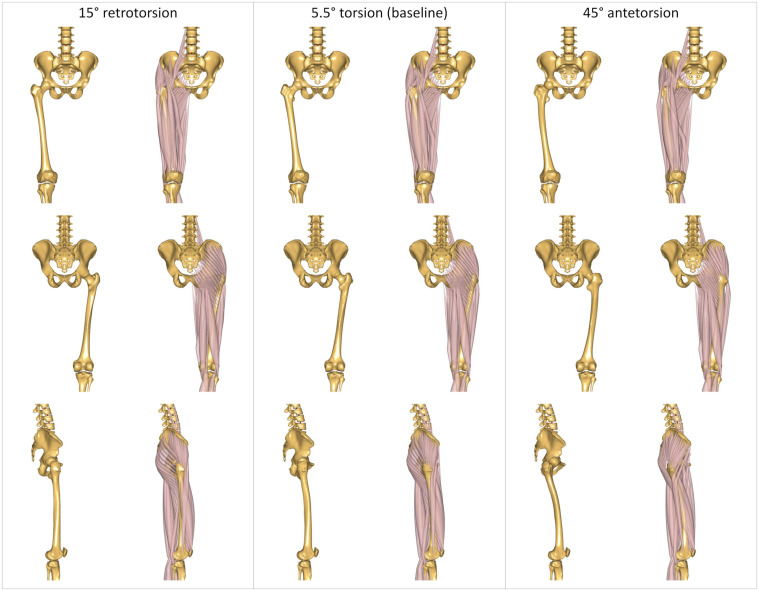
Three representative modeled torsional configurations implemented in the AnyBody Modeling System. The femurs are characterized by 15° of retrotorsion (left); 5.5° of antetorsion (center), corresponding to the baseline unmorphed model; and 45° of antetorsion (right). Illustrations report anterior (top), posterior (middle), and lateral (bottom) views. Each view is reported without and with muscles, to better visualize the effect of the morphing on the bone geometry, and the resulting changes in the muscles’ lines of action.

The morphed femurs present different orientations of the muscles’ line of actions relative to the joints’ positions, as illustrated in Figure 2 for three different torsional configurations.

The lever arms of the femur-spanning muscles were also qualitatively evaluated for four arbitrary modeled femoral morphologies: The baseline unmorphed model, characterized by a femoral antetorsion of 5.5°, as well as morphed femurs with retrotorsion of –15° and antetorsion of + 25° and + 45°. These femoral morphologies were implemented in a generic model corresponding to the 50th percentile male anthropometrics. Lever arms were calculated for each individual muscle fascicle across artificial ranges of hip sagittal motion (20° extension, 90° flexion), hip frontal motion (30° adduction, 50° abduction), and hip transversal motion (40° internal, 40° external rotation). Average lever arms were calculated for the fascicles constituting each muscle over the different joint ranges of motion (De Pieri et al., 2018). Additionally, muscle lever arms were also calculated for the morphed femur with + 45° of antetorsion and a fixed hip internal rotation angle of 20°, in order to mimic a compensatory kinematic strategy suspected to restore abduction capacity in pathological patients with torsional deformities (Arnold et al., 1997).

### Gait Analysis

Gait trials were processed and analyzed through the toolkit AnyPyTools^1^ (Lund et al., 2019), in the Python programming language (Python Software Foundation). 3D hip kinematics were calculated in the anatomical coordinate systems described by Klein Horsman et al. (2007) and based on the International Society for Biomechanics’ (ISB) recommendations (Wu et al., 2002). The foot progression angle relative to the direction of gait was also calculated. The orientation of the foot was identified through an axis connecting the heel and the second-metatarsal markers, while the direction of gait was defined as the line connecting the positions of the heel marker in two consecutive ipsilateral heel strikes. 3D hip internal net moments (or torques) were reported in the proximal coordinate system (pelvis) according to ISB recommendations (Wu et al., 2002; Derrick et al., 2020). Joint moments were normalized by body mass.

All the data were time-normalized from heel strike (0%) to heel strike (100%) and interpolated to 1% steps (101 points). An average per patient was then calculated based on the five gait trials collected.

### Muscle Forces and HCFs

The optimal configuration of muscle forces necessary for the generation of the overall lower-limb joint torques were computed based on the muscle recruitment criterion. The reported muscle forces are defined as the sum of the forces generated by all fascicles constituting each muscle.

**FIGURE 3:**
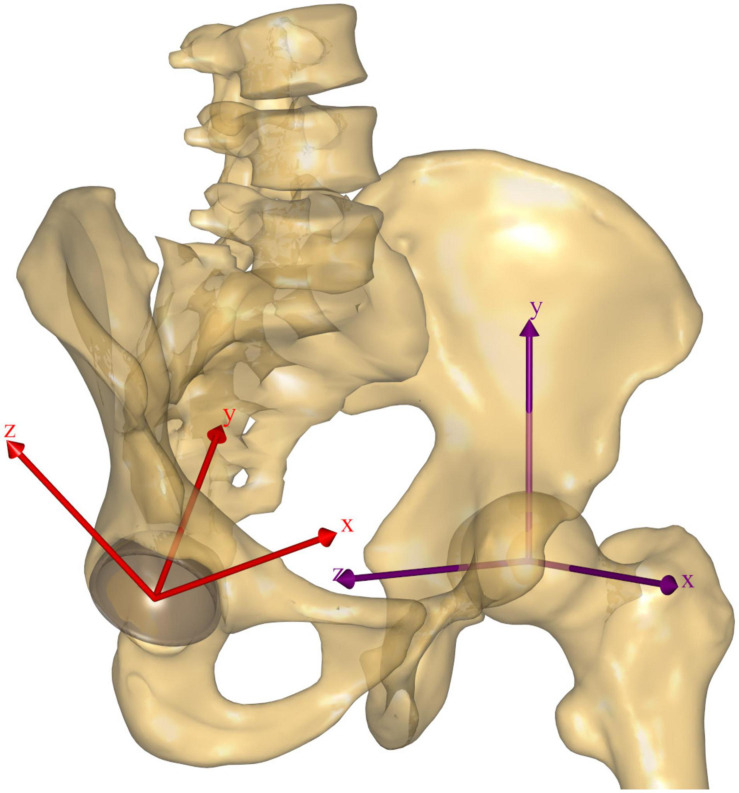
The pelvis-based reference frame according to ISB recommendations is reported in purple for the left hip, while the acetabular reference frame is reported in red for the right hip. The latter is aligned with the acetabular opening plane for the decomposition of the HCF vector; its origin is coincident with the HJC, the *y* axis is perpendicular to the acetabular opening plane, and the *x* axis is oriented to the anterior (+)/posterior (–) direction, while the *z* axis is oriented to the superolateral direction (+) and passes through the mid-acetabular notch (–). The idealized hemisphere used to calculate the intersection of the HCF vector on the acetabulum is illustrated for the right hip in light gray.

Resulting HCFs were also calculated in a proximal (pelvis-based) coordinate system according to ISB recommendations (Wu et al., 2002; Derrick et al., 2020; Figure 3).

Muscle forces and HCFs were normalized by body weight, time-normalized from heel strike to heel strike, and averaged per patient.

Additionally, to estimate the orientation of the HCF on the acetabulum, the HCF vector was intersected with an idealized hemisphere representing the acetabulum, and the contact force pathways were plotted (Weber et al., 2012; De Pieri et al., 2020). The components of the HCF vector were decomposed in a reference frame with its origin in the HJC and aligned with the acetabular opening plane, standardized to 45° of inclination and 20° of anteversion for all subjects, as shown in Figure 3. Positive x components indicate anteriorly oriented forces, while positive z components indicate superolateral oriented forces. The diameter of the hemisphere was also standardized to 54 mm (Krebs et al., 2009). Average force contact pathways were calculated for each subject and for the mean HCF vector across the whole cohort.

### Statistical Parametric Mapping Analysis

The relationships between femoral torsion and lower-limb kinematics, hip internal net moments, HCFs, and muscle forces were analyzed by means of statistical parametric mapping (SPM) (Friston et al., 1994; SPM1d^2^, v0.4.3; Pataky, 2012).

The three kinematic components of the hip joint were regarded as a vector field, describing the 3D variation over time of the kinematic vector trajectory (Pataky et al., 2013), while the foot progression angle was considered as separate one-dimensional, time-dependent scalar. Canonical correlation analyses (CCAs), the vectorial equivalent of a linear regression, were carried out to evaluate the effect of femoral torsion on hip kinematics in the examined cohort. The use of vector field analysis takes into consideration covariance between spatial components, thus reducing errors due to covariation bias (Pataky et al., 2013). Statistically significant correlation between femoral torsion and foot progression angle was instead analyzed through a scalar linear regression analysis.

CCAs were also conducted to identify statistically significant correlations between femoral torsion and hip internal net moments and HCFs, both described as 3D vectorial fields. Additionally, muscles were grouped according to their main function in hip extensors, hip flexors, hip abductors, and hip adductors. The forces generated by the muscles in each functional group were also considered as multidimensional vectorial field, similar to Pataky et al. (2013). CCA was also used to analyze the relationships between femoral torsion and the forces generated in each functional muscle compartment.

The relevant output test statistic—SPM{X 2} for CCA and SPM{t} for linear regression—was evaluated at each point of the gait cycle (GC). The significance level was set at α = 0.05, and the corresponding critical thresholds—X2^∗^ or t^∗^—were calculated based on the temporal smoothness of the input data through random field theory. Finally, the probability that similar suprathreshold regions would have occurred from equally smooth random waveforms was calculated. In case of vectorial CCA, *post hoc* scalar field linear regressions were also conducted separately on each component of the vectorial field, with Bonferroni-corrected significance threshold levels, adjusted at α = 0.05/*n*, with *n* indicating the number of components of the specific vectorial field.

In the interest of clarity, only differences which were statistically significant for more than 2% of GC are discussed.

### Effect of Modeling Femoral Torsion

Finally, the effect of including subject-specific femoral torsion in the models was evaluated by comparing the predictions of the models with generic or personalized femoral morphologies. Specifically, an SPM vectorial paired Hotelling *T*^2^ test was conducted to investigate differences in 3D HCFs, with a significance level of α = 0.05. *Post hoc* scalar field linear paired *t*-tests were also conducted separately on each force component. The root mean square deviation (RMSD) between HCF components predicted with the generic or personalized models was also calculated for each subject and reported against subject-specific femoral torsion values.

## Results

### Muscle Lever Arms

The qualitative analysis of muscle lever arms indicates a reduction of iliacus and rectus femoris hip flexing lever arm with increased antetorsion values, particularly when the hip is in an extended position, as well as changes in iliacus, psoas major, and rectus hip external/internal rotation lever arms.

The superior compartment of the gluteus maximus presents reduced lever arms for hip extension with a retrotorted femur, while both its extensive and abductive lever arms are increased for antetorted morphologies.

**FIGURE 4:**
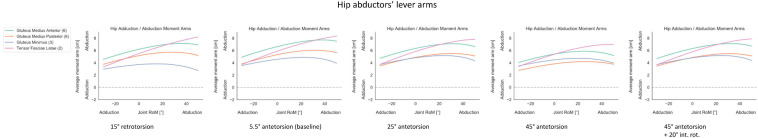
Hip abductors’ lever arms were calculated for each individual muscle fascicle across a range of hip frontal motion (30° adduction, 50° abduction). Average lever arms were calculated for the fascicles constituting each muscle and the specific number of fascicles is reported in brackets next to the muscle’s name. Four different modelled femoral morphologies were compared: the baseline unmorphed model, characterized by a femoral antetorsion of 5.5°, as well as morphed femurs with retrotorsion of −15° and antetorsion of +25° and +45°, reported as columns. Additionally, muscle lever arms were also calculated for the morphed femur with +45° of ante-torsion and a fixed hip internal rotation angle of 20° (rightmost column), in order to mimic a compensatory kinematic strategy suspected to restore abduction capacity in pathological patients with torsional deformities. A complete overview of the changes in muscle lever arms is reported in Supplementary Figure 1.

The abductor muscles see a decrease in their abductive lever arms with antetorted morphologies; however, the convenience of their lever arms is restored when an additional, fixed, 20° internal rotation of the hip is modeled (Figure 4). Gluteus medius and minimus also acquire a more convenient extensive lever arm for increased antetorsion values, while their internal/external rotation lever arms are affected in both retrotorted and antetorted configurations.

The lever arms around the hip of the adductor muscles are relatively unaffected by the modeling of different femoral morphologies.

A complete overview of the changes in muscle lever arms for different modeled femoral morphologies is reported as Supplementary Figure 1.

### Gait Kinematics and Kinetics

**FIGURE 5:**
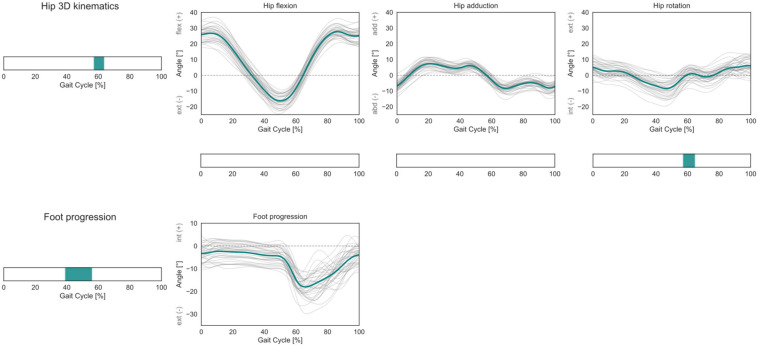
Hip 3D kinematics and foot progression angle over the GC. Average profiles for each subject are reported in gray, while the cohort’s mean is reported in turquoise. For the hip joint 3D kinematics, phases of GC in which a statistically significant correlation was observed in the SPM vector-field CCA are indicated in the leftmost panel. Below each kinematic component, the results of the *post hoc* scalar-field linear regression analyses are reported. For the foot progression angle, the phases of GC in which a statistically significant correlation was observed in the SPM scalar-field linear regression analysis are indicated in the leftmost panel.

Within the investigated cohort, statistically significant correlations between femoral torsion angles and the subjects’ kinematics during gait were observed for the 3D hip joint angles during pre-swing to initial swing (57–63% GC). The *post hoc* linear regression analysis of the three individual kinematic components indicates a negative correlation between femoral torsion and hip external rotation. The foot progression angle presents a positive correlation with femoral torsion during terminal stance (39–56% GC) (Figure 5).

**FIGURE 6:**
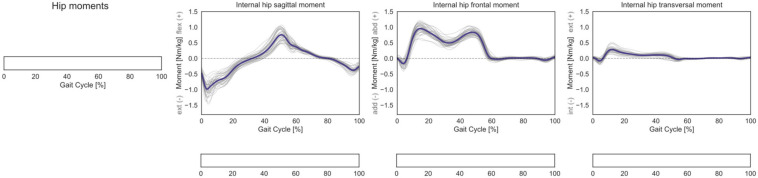
Hip 3D internal net joint moments over the GC. Moments are normalized by body mass. Average profiles for each subject are reported in gray, while the cohort’s mean is reported in dark blue. No statistically significant correlation was observed in the SPM vector-field CCA (leftmost panel), nor in the *post hoc* scalar-field linear regression analyses (below each kinematic component).

No statistically significant correlation was observed for the 3D hip internal joint moments in the investigated cohort (Figure 6).

### Muscle Forces and HCFs

**FIGURE 7:**
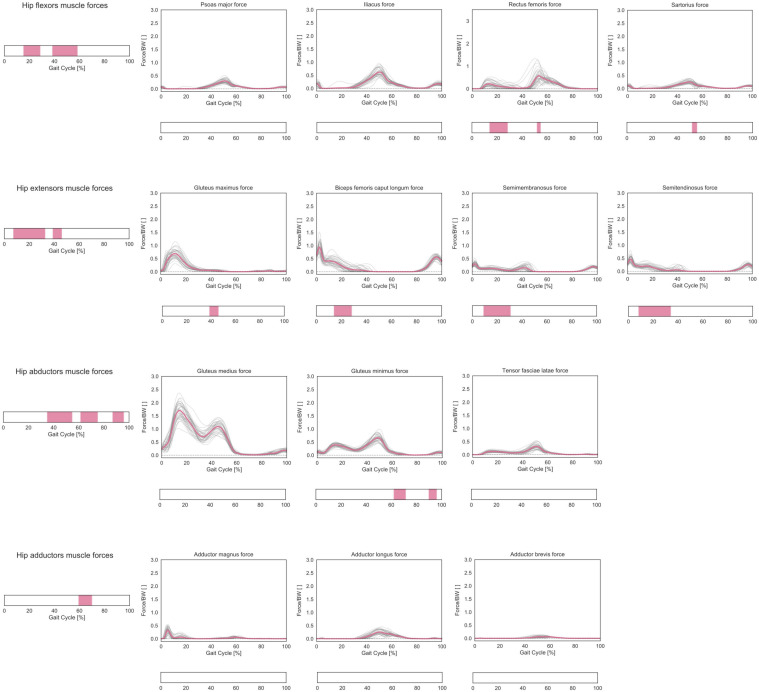
Predicted lower-limb muscle forces during the GC. Forces are normalized by body weight (BW). Average profiles for each subject are reported in gray, while the cohort’s mean is reported in pink. Muscles were grouped according to their main function in hip extensors, hip flexors, hip abductors, and hip adductors, and the predicted muscle forces in each functional group were considered as a multidimensional vectorial field. For each muscle compartment, phases of GC in which a statistically significant correlation was observed in the SPM vector-field CCA are indicated in the leftmost panel. Below each muscle, the results of the *post hoc* scalar-field linear regression analyses are reported.

In terms of predicted muscle forces required during gait, correlations were found for hip flexors, extensors, abductors, and adductors muscle groups (Figure 7). The forces generated by the hip flexors correlated with femoral torsion during mid-stance and terminal stance (15–28% and 38–58% GC, respectively). The *post hoc* analysis revealed a positive correlation for the rectus femoris during mid-stance and for both rectus femoris and sartorius during terminal stance. The forces generated by the hip extensors correlated with femoral torsion from loading response to mid-stance and during terminal stance (7–33% and 39–46% GC). The *post hoc* analysis revealed a positive correlation for the gluteus maximus during terminal stance and a negative correlation for biceps femoris, semimembranosus, and semitendinosus during mid-stance. The forces generated by the hip abductors correlated with femoral torsion during terminal stance, initial swing, and terminal swing (35–55%, 61–75%, and 87–96% GC, respectively). The *post hoc* analysis revealed a negative correlation for the gluteus minimus during initial and terminal swing phases. The forces generated by the hip adductors correlated with femoral torsion during the initial swing phase (59–70% GC). The *post hoc* analysis did not reveal any prominent trend for the individual adductor muscles.

**FIGURE 8:**
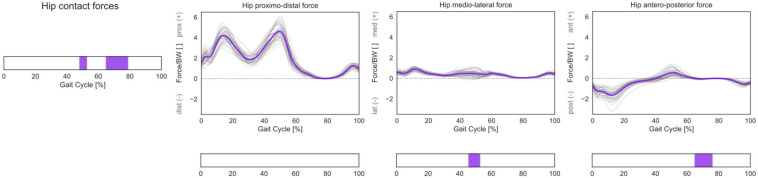
3D HCFs over the GC. HCFs were calculated in a proximal (pelvis-based) coordinate system according to ISB recommendations and are normalized by body weight (BW). Average profiles for each subject are reported in gray, while the cohort’s mean is reported in violet. Phases of GC in which a statistically significant correlation was observed in the SPM vector-field CCA are indicated in the leftmost panel. Below each force component, the results of the *post hoc* scalar-field linear regression analyses are reported.

A statistically significant correlation was found between 3D HCFs and femoral torsion in the investigated cohort during terminal stance and mid-swing (48–52% and 65–79% GC). From the *post hoc* analysis, it emerged that subjects with higher antetorsion have more medially oriented HCFs in mid-stance and more anteriorly oriented HCFs during the swing phase (Figure 8).

A more extensive analysis of all SPM output test statistics is reported in Supplementary Figure 2.

**FIGURE 9:**
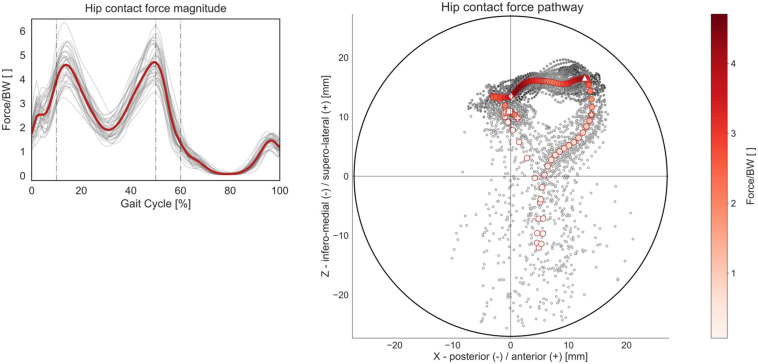
HCF magnitude and acetabular pathway over the GC. Average profiles for each subject are reported in gray, while the cohort’s mean is reported in red. Forces are reported normalized by body weight (BW). The HCF pathway was calculated as the intersection of the HCF vector with a hemisphere fitting the acetabulum, standardized to a diameter of 54 mm. Each circular marker represents one of the 101 time points in which the GC was discretized. The initial heel strike (0% GC) is highlighted with a squared marker, the transition from initial loading response to mid-stance (10% GC) with a diamond marker, the transition from terminal stance to pre-swing (50% GC) with a triangular marker, the transition from stance to swing phase (60% GC) with a star marker, and the final heel strike (100% GC) with a cross marker.

The qualitative analysis of the mean HCF pathway reveals that the loads during gait are transmitted from the femur mostly to the anterior superolateral quadrant of the acetabulum (Figure 9). During the initial loading response (0–10% GC), the HCF vector is slightly posteriorly oriented in the upper half of the acetabulum, while during mid-stance and terminal stance (10–50% GC), corresponding to single-limb support, the HCF vector shifts more anteriorly and is characterized by higher values in terms of magnitude. During pre-swing (50–60% GC), the HCF vector starts translating inferomedially while maintaining an overall anterior orientation. During the swing phase, the intra-articular loads transmitted through the hip are smaller, and the HCF vector produces a longer contact path on the acetabulum, starting from the anterior/superolateral quarter, spanning through the anterior/inferomedial one, and ending in the center of the superolateral half.

### Effect of Modeling Femoral Torsion

**FIGURE 10:**
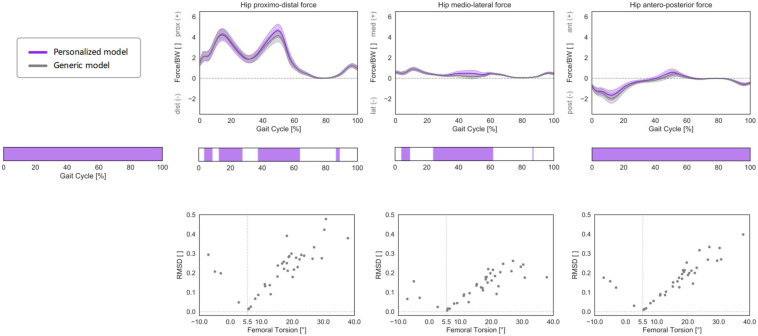
3D HCFs (mean ± standard deviation) over the GC computed with a generic, baseline musculoskeletal model (in gray) or with a personalized model accounting for subject-specific femoral torsion (in violet). HCFs were calculated in a proximal (pelvis-based) coordinate system according to ISB recommendations and are normalized by body weight (BW). Phases of GC in which a statistically significant correlation was observed in the SPM vectorial paired Hotelling *T*^2^ are indicated in the leftmost panel. Below each force component, the results of the *post hoc* scalar-field paired *t*-tests are reported. Root mean standard deviation between the HCFs predicted with the two different models was calculated for each subject and plotted against subject-specific femoral torsion values. The torsional value of the baseline model geometry (5.5°) is reported as a dashed vertical line. Positive values indicate antetorsion, negative retrotorsion.

The inclusion of a morphed, personalized femoral torsional morphology in the models led to statistically significant differences in HCFs through the entire GC when compared to models with a generic (baseline) femoral morphology (Figure 10). In particular, the models with morphed femoral geometry predicted less proximally and medially oriented forces during mid-stance to terminal stance and more anteriorly oriented forces throughout the GC. The RMSD between HCF components predicted with generic or personalized models increased with a qualitative linear trend for larger deviations between subject-specific femoral torsion and the torsion of the baseline generic model (5.5°), for both antetorted and retrotorted configurations.

## Discussion

This study investigated the effect of femoral torsion on hip kinematics, kinetics, muscle forces, and contact forces during gait in a group of asymptomatic adults presenting a heterogeneous range of femoral torsion. For this purpose, personalized musculoskeletal models were created based on individual morphological data obtained with low-dosage radiography and driven with matching individual kinematic data acquired during motion-capture gait analysis. HCFs predicted through musculoskeletal models were compared against the predictions obtained with a generic model, and the changes in muscle lever arms associated with different degrees of femoral torsion were also qualitatively analyzed.

Within the investigated cohort, higher femoral antetorsion led to significantly higher anteromedial HCFs during gait (medial during loaded stance phase and anterior during swing phase). Additionally, statistically significant correlations with femoral torsion were observed for foot and hip kinematics. In particular, subjects with higher antetorsion walked with a more internally rotated foot during terminal stance and with a more internally rotated hip in the transition from stance to swing. This could indicate that specific kinematic patterns or compensatory mechanisms could be adopted also in the asymptomatic population. This result indicates that a complete evaluation of a condition must include a functional assessment of a subject/patient and specifically take into account his/her specific joint kinematics.

The analysis of the muscles’ lever arms in different modeled femoral morphologies indicates that femoral antetorsion has an important effect on the lever arm length of several muscles and particularly on the abductive capacity of gluteus medius and gluteus minimus. Similar to that of Arnold et al. (1997), this study confirmed that internally rotating the hip can restore the hip abductor lever arms in the presence of excessive femoral antetorsion. Hip abductors have an important functional role in the stability of the hip and pelvis (Retchford et al., 2013), particularly during gait (van der Krogt et al., 2012). Muscle weakness of the hip abductors may result in compensatory activation of other muscles (van der Krogt et al., 2012) and lead to more anterior HCFs (Lewis et al., 2007). Moreover, muscle weaknesses in the presence of altered femoral morphology can further impair gait performance (Vandekerckhove et al., 2021). In the examined cohort, statistically significant correlations between femoral torsion and the muscle forces generated during gait by hip flexor, extensor, abductor, and adductor muscle groups were also observed. Femoral antetorsion, through altered kinematic strategies and/or different muscle activations and forces, may therefore lead to altered HCFs and pose a risk for articular damage.

The personalization of the musculoskeletal models based on subject-specific torsional values led to statistically significant differences in the predicted HCFs throughout the GC in comparison to the generic baseline model, based on a cadaveric template. This finding is in agreement with previous studies (Passmore et al., 2018; Kainz et al., 2020). Higher RMSD values were found for subjects with high femoral antetorsion or high femoral retrotorsion, indicating that the more a subject’s morphology differs from the generic model, the more important it is to account for these differences. While a fully subject-specific modeling approach would require the inclusion of bone geometries and muscle lines of actions from CT scans or MRI (Dejtiar et al., 2020; Modenese and Kohout, 2020), it is also a rather time-consuming approach (Andersen, 2021), and it could introduce additional uncertainties and errors in the identification of muscle insertion and origins (Carbone et al., 2012; Valente et al., 2014).

Morphing the femoral geometry of a generic model to match the torsional angle of a specific subject represents a rapid and effective alternative to personalized models, which could therefore be more applicable in a clinical setting. Using nominal torsional values as input, musculoskeletal models could also be personalized when the acquisition of imaging data is not possible and only clinical assessments of the torsional angles are available (Scorcelletti et al., 2020), even if these measurements are characterized by a larger uncertainty (Günther et al., 1996).

The mean HCF pathway revealed that loads during gait are transmitted from the femur mostly to the anterior superolateral quadrant of the acetabulum. While the qualitative analysis of the HCF pathway in this study was based on a generic acetabular orientation, including subject-specific information on acetabular inclination and anteversion might help to accurately identify whether certain subjects present high loads applied onto specific peripheral regions of the acetabulum. The clinical relevance of analyzing intra-articular load distributions in patients with hip pathologies remains to be further verified and would certainly require a more comprehensive analysis of all other anatomical factors that could predispose to hip pathologies. For instance, in patients with FAI syndrome, cartilage degeneration occurs mainly in the anterosuperior portion of the acetabulum (Beck et al., 2005). This was confirmed through a large multicenter observational study (Pascual-Garrido et al., 2019), which reported a high incidence of both anterior and superolateral peripheral cartilage lesions. A more anterior or more superolaterally oriented HCF may induce higher stresses in the more peripheral regions, thus accelerating cartilage degeneration. Finite elements analyses accounting for pathological hip anatomies, altered contact mechanics, and patient-specific loads might represent a more accurate tool to investigate the localized stresses that occur within the joint in the presence of FAI syndrome (Ng et al., 2016, 2017, 2018).

This study investigated the effect of a single alignment parameter such as femoral torsion on lower-limb function and specifically on hip loads. However, several other anatomical and morphological parameters could affect the mechanics of the hip. Anatomical variations in neck-shaft angle could similarly alter the relative alignment of the muscles around the joint and therefore affect the resulting HCFs (Kainz et al., 2020). Other parameters such as acetabular coverage, acetabular retroversion, and presence of cam/pincer deformities would not have a direct effect on hip mechanics in our models, given the assumption of a perfect ball-and-socket hip joint. Nevertheless, these factors, especially when pathological, could influence the overall kinematics and kinetics of the affected subjects, for instance, through pain-avoiding strategies, and this could also influence the models’ outcome.

The study was limited to 37 healthy individuals. The analysis of asymptomatic adults is not affected by symptoms and impairments that patients could experience, thus potentially providing an insight on the contribution of femoral torsion alone to the loading of the hip joint. No other anatomical parameter related to femoral or acetabular morphology was analyzed for the investigated subjects; therefore, it cannot be excluded that any of them presented an unknown pathological anatomy, which could have biased the results of this study. However, participants presenting pain in the lower back or in the lower extremities were excluded from the study, thus potentially excluding other severe (and symptomatic) pathoanatomies. Moreover, the recruited participants were all rather young, fit, and active and therefore do not represent the general population. Subject demographics, such as age, BMI, and sex, have been previously shown to relate to HCFs (De Pieri et al., 2019; Lunn et al., 2020). We did not find any statistically significant correlation between femoral torsion values and age or BMI in our investigated cohort, nor did we find a statistically significant difference between torsional values of males and females. While this does not indicate any clear bias in the selection of our cohort, a multivariate analysis based on a larger sample size would be required to exclude the effect of any other confounding factor, in terms of both demographics and anatomical variability. Additionally, one randomly chosen leg for each subject was included in the study. While other selection strategies, such as the more antetorted or dominant leg, could have led to different results, a left/right randomization was considered the most conservative option to avoid introducing any unknown bias, for instance, associated with other morphological factors that were not considered in this study.

Future work should aim at including symptomatic patients with excessive femoral antetorsion, which might present more pronounced kinematic compensatory mechanisms, such as in-toeing, as well as protective strategies to avoid pain (Ng et al., 2018). The qualitative evaluation of the muscle lever arms suggested that the effect of these torsional deformities might be more pronounced for pathological ranges of femoral torsion, when the moment-generating capacity of the abductor muscles is substantially reduced. This study was also limited to an analysis of gait, which represents the most commonly performed daily activity (Morlock et al., 2001); however, musculoskeletal modeling can be used to accurately assess HCFs during various activities of daily living (De Pieri et al., 2019; Lunn et al., 2020), in which patients with hip pathologies could present more physical impairments (Diamond et al., 2015; Kierkegaard et al., 2017).

Excessive femoral torsion can affect both hip and knee mechanics (Passmore et al., 2018) and is associated with functional impairments and motor limitations (Bruderer-Hofstetter et al., 2015). Torsional deformities of the femur and tibia (Bruce and Stevens, 2004; Alexander et al., 2020) have also been associated with knee and patellar complications (Eckhoff et al., 1997; Powers, 2003; Stevens et al., 2014; Bretin et al., 2011). However, a better understanding of the femorotibial and femoropatellar joint mechanics, specifically of their response to torsional forces, would require a more complex multi-DOF modeling approach of the knee complex, accounting for specific morphological variations of the tibia and femur, specifically of their articulating surfaces, as well as passive soft-tissue structures which constrain knee rotations (Marra et al., 2014; Lenhart et al., 2015; Dejtiar et al., 2020).

## Conclusion

The method proposed in this study, which accounts for both morphological and kinematic characteristics, can serve as a blueprint for a structured investigation of alterations in hip intra-articular loads as a result of altered femoral torsional alignment.

The use of low-dosage imaging techniques combined with gait analysis and musculoskeletal modeling might help in identifying in a clinical setting patients who could be at a higher risk for cartilage damage and early onset of hip OA due to femoral torsion alone. Additionally, it could help in identifying among patients with excessive femoral torsion those who present more severe functional impairments as a consequence of altered joint mechanics. Long-term monitoring of pediatric and adolescent patients might also provide a better understanding of the long-term effects of torsional abnormalities on joint intra-articular loads and cartilage health.

While this study investigated the effect of femoral torsion alone, the proposed method should be extended to account for all femoral and acetabular morphological variations that could lead to altered and pathological hip mechanics. A better understanding of the forces produced within the acetabulum and of the mechanical consequences of the overall limb alignment is necessary to improve individual diagnoses and to optimally plan targeted bone corrective surgeries of the lower extremities, which could reduce the long-term risk of (over-)loading-induced joint degeneration.

## Data Availability Statement

The datasets presented in this article are not readily available because of privacy restrictions. Requests to access the datasets should be directed to SF.

## Ethics Statement

The studies involving human participants were reviewed and approved by Ethikkommission Zürich (BASEC-Nr. 2019-00688). The patients/participants provided their written informed consent to participate in this study. Written informed consent was obtained from the individual(s) for the publication of any potentially identifiable images or data included in this article.

## Author Contributions

ED contributed to study conceptualization, study design, method development, software, data curation, data visualization, data analysis, data interpretation, and writing. BF contributed to study conceptualization, study design, ethics, method development, data acquisition, data interpretation, and writing. RL contributed to study design, method development, data acquisition, data interpretation, and writing. SM contributed to method development, data acquisition, data curation, and writing. NC contributed to study design, method development, data interpretation, and writing. ML and SF contributed to study conceptualization, study design, ethics, data interpretation, and writing. All authors contributed to the article and approved the submitted version.

## Conflict of Interest

The authors declare that the research was conducted in the absence of any commercial or financial relationships that could be construed as a potential conflict of interest.

## Footnotes

1 https://github.com/AnyBody-Research-Group/AnyPyTools

2 www.spm1d.org
