# Supplementary material for: Subject-Specific Modeling of Femoral Torsion Influences the Prediction of Hip Loading During Gait in Asymptomatic Adults
Source: Front Bioeng Biotechnol. 2021 Jul 21;9:679360. doi: 10.3389/fbioe.2021.679360 (PMC8334869; doi:10.3389/fbioe.2021.679360)
Supplement: Supplementary file 2 [file Data_Sheet_2.PDF]

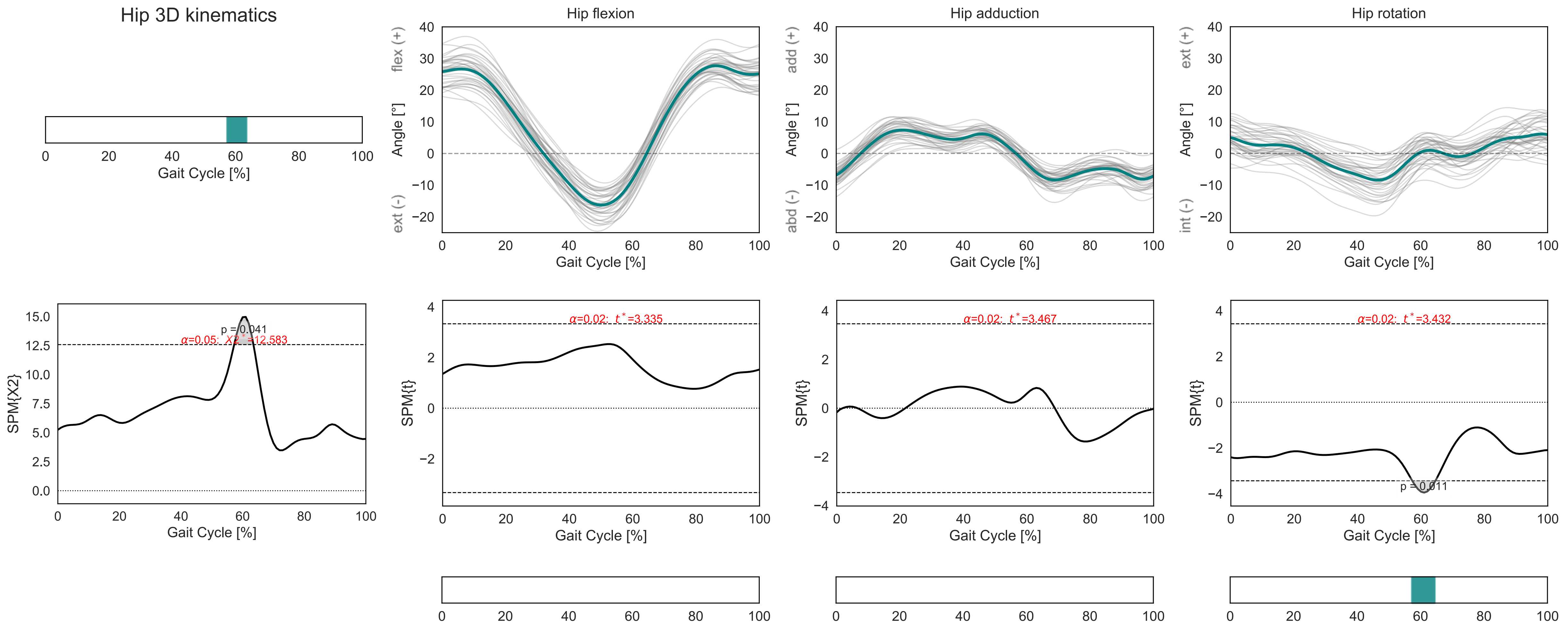
Hip 3D kinematics
Hip flexion
Hip adduction
Hip rotation
Gait Cycle [%]
Angle [°]
flex (+)
ext (-)
add (+)
abd (-)
ext (+)
int (-)
SPM{X2}
SPM{t}
p = 0.041
α=0.05: X2*=12.583
α=0.02: t*=3.335
α=0.02: t*=3.467
α=0.02: t*=3.432
p = 0.011

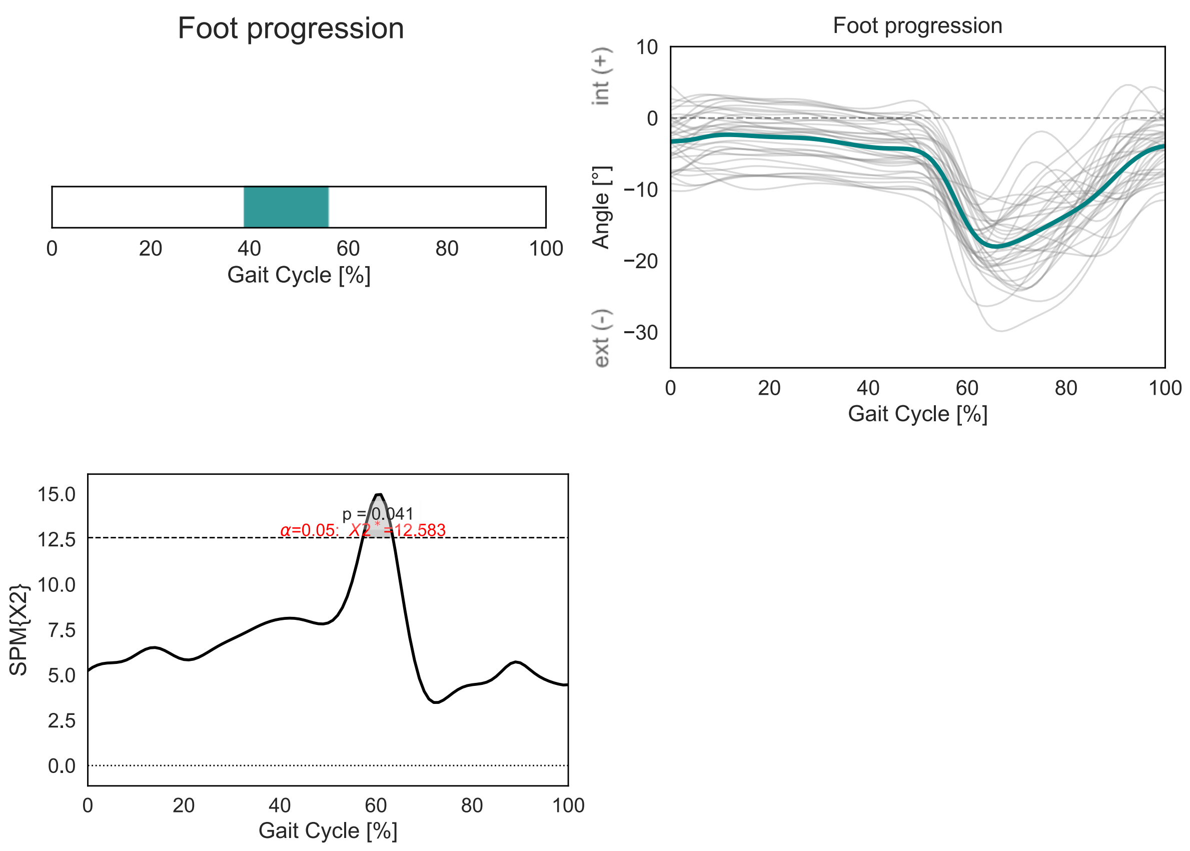
Foot progression
Foot progression
Gait Cycle [%]
Angle [°]
int (+)
ext (-)
SPM{X2}
p = 0.041
α=0.05: X2*=12.583

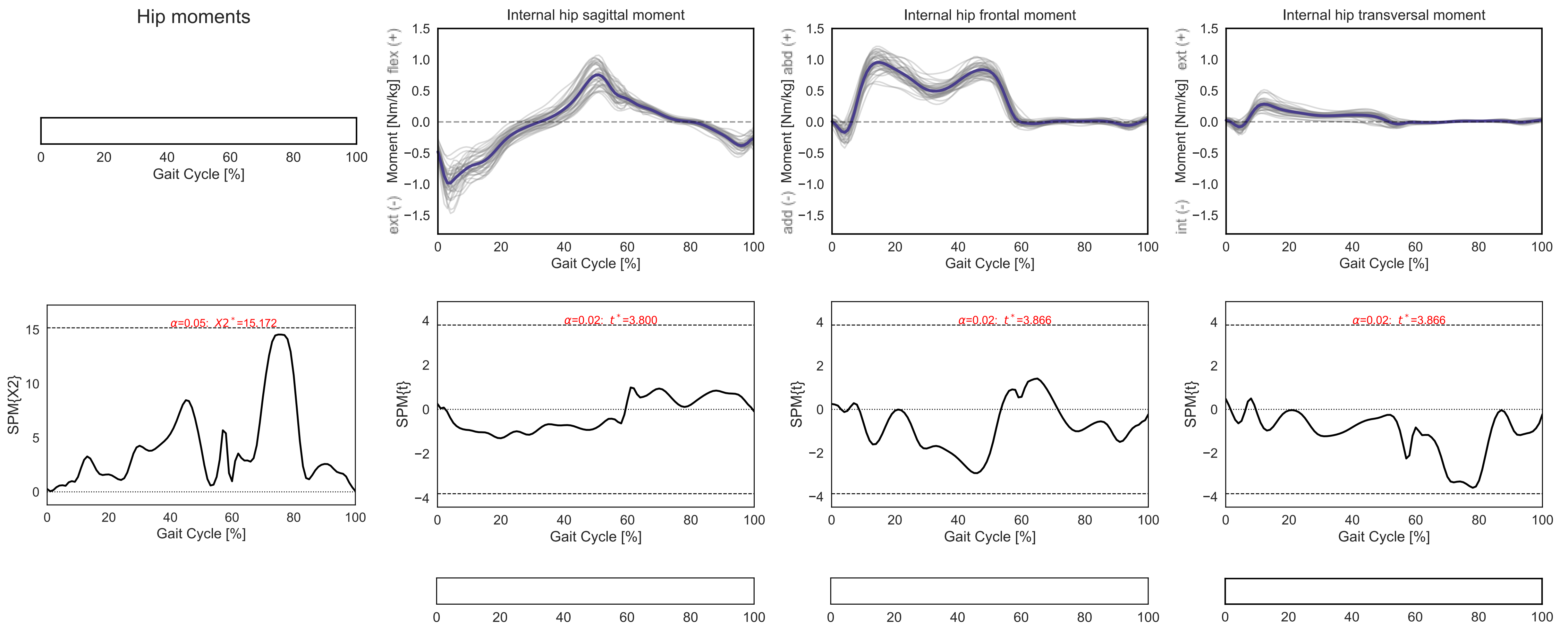
Hip moments
Internal hip sagittal moment
Internal hip frontal moment
Internal hip transversal moment
Gait Cycle [%]
Moment [Nm/kg]
flex (+)
ext (-)
abd (+)
add (-)
ext (+)
int (-)
SPM{X2}
SPM{t}
α=0.05: X2*=15.172
α=0.02: t*=3.800
α=0.02: t*=3.866
α=0.02: t*=3.866

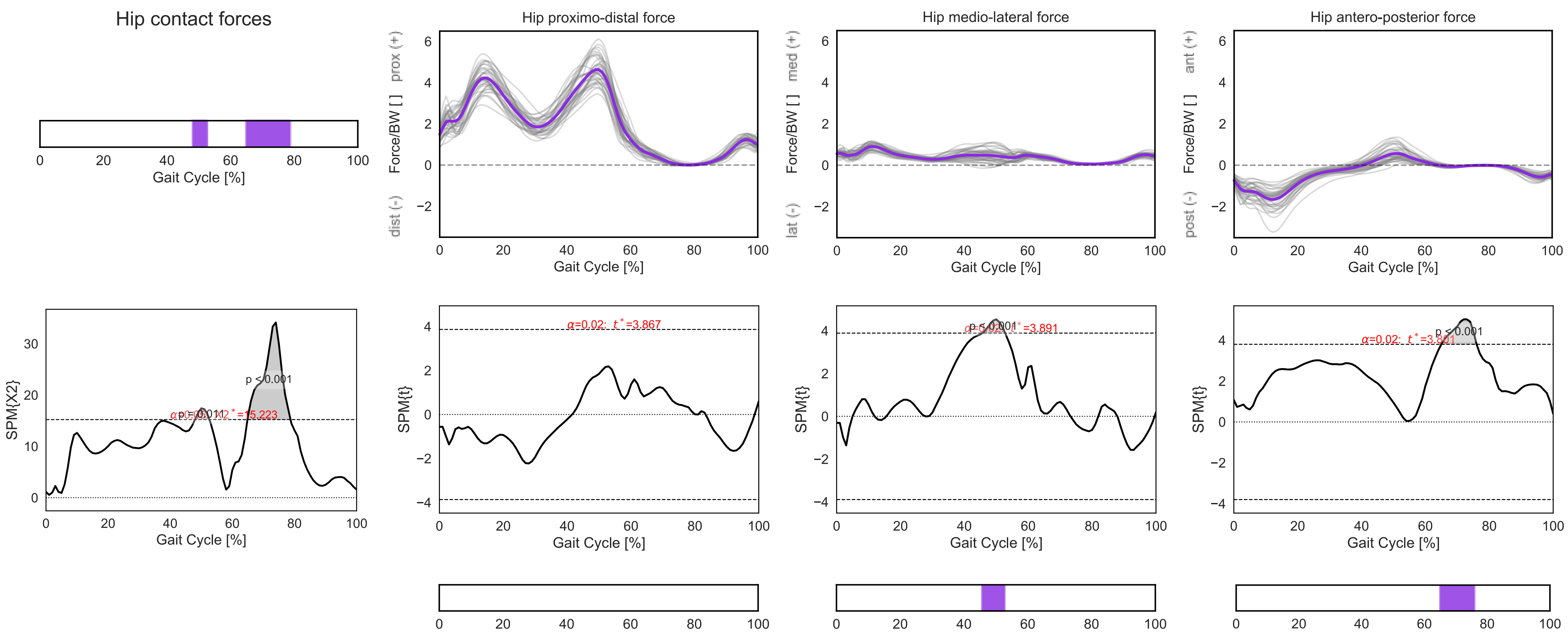
Hip contact forces
Hip proximo-distal force
Hip medio-lateral force
Hip antero-posterior force
Gait Cycle [%]
Force/BW [ ]
prox (+)
dist (-)
med (+)
lat (-)
ant (+)
post (-)
SPM{X2}
SPM{t}
p < 0.001
α=0.05: X2*=15.223
α=0.02: t*=3.867
α=0.02: t*=3.891
α=0.02: t*=3.861

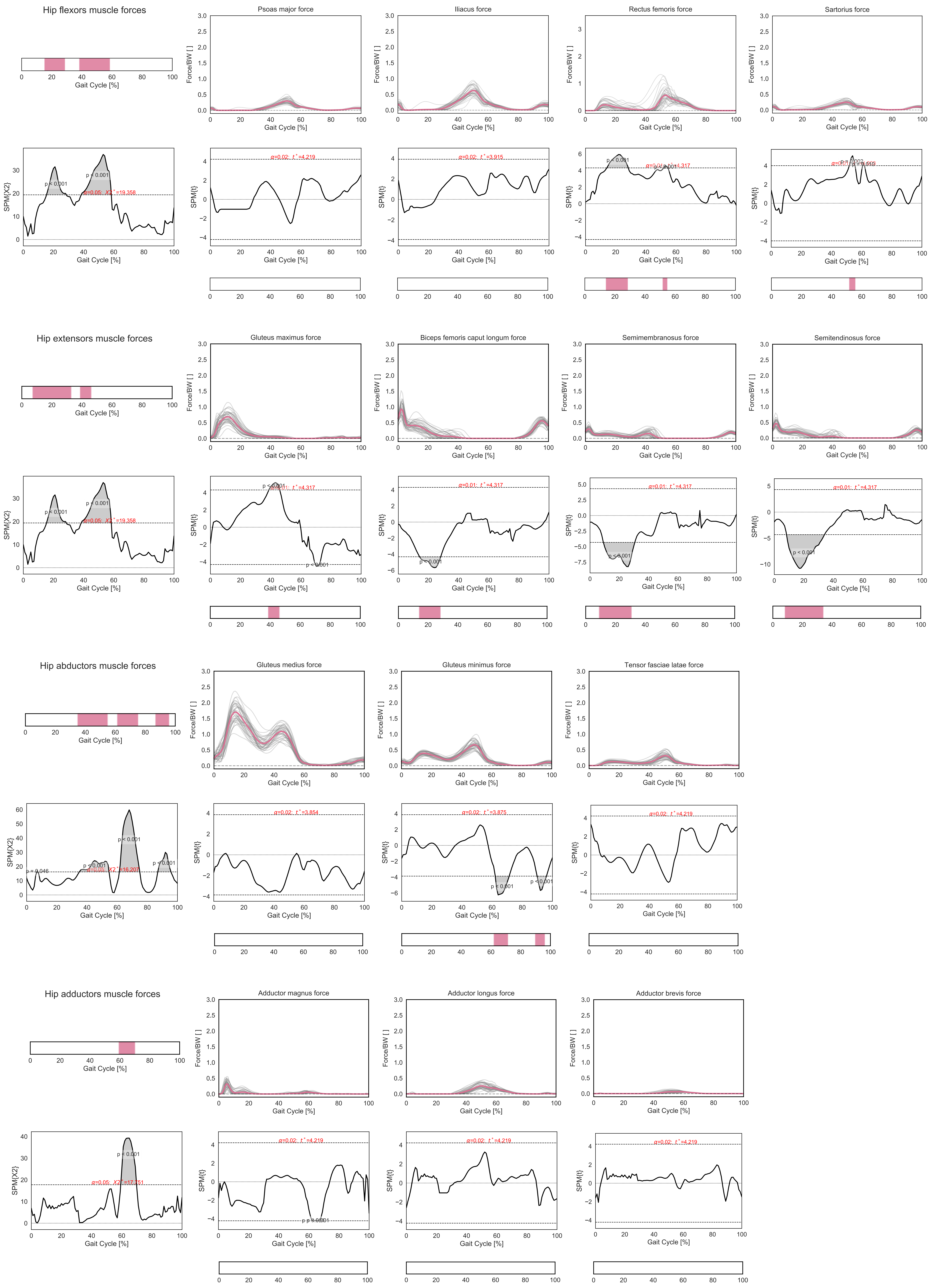

Hip flexors muscle forces
Psoas major force
Iliacus force
Rectus femoris force
Sartorius force
Hip extensors muscle forces
Gluteus maximus force
Biceps femoris caput longum force
Semimembranosus force
Semitendinosus force
Hip abductors muscle forces
Gluteus medius force
Gluteus minimus force
Tensor fasciae latae force
Hip adductors muscle forces
Adductor magnus force
Adductor longus force
Adductor brevis force
Gait Cycle [%]
Force/BW [ ]
SPM{t}
SPM{X2}

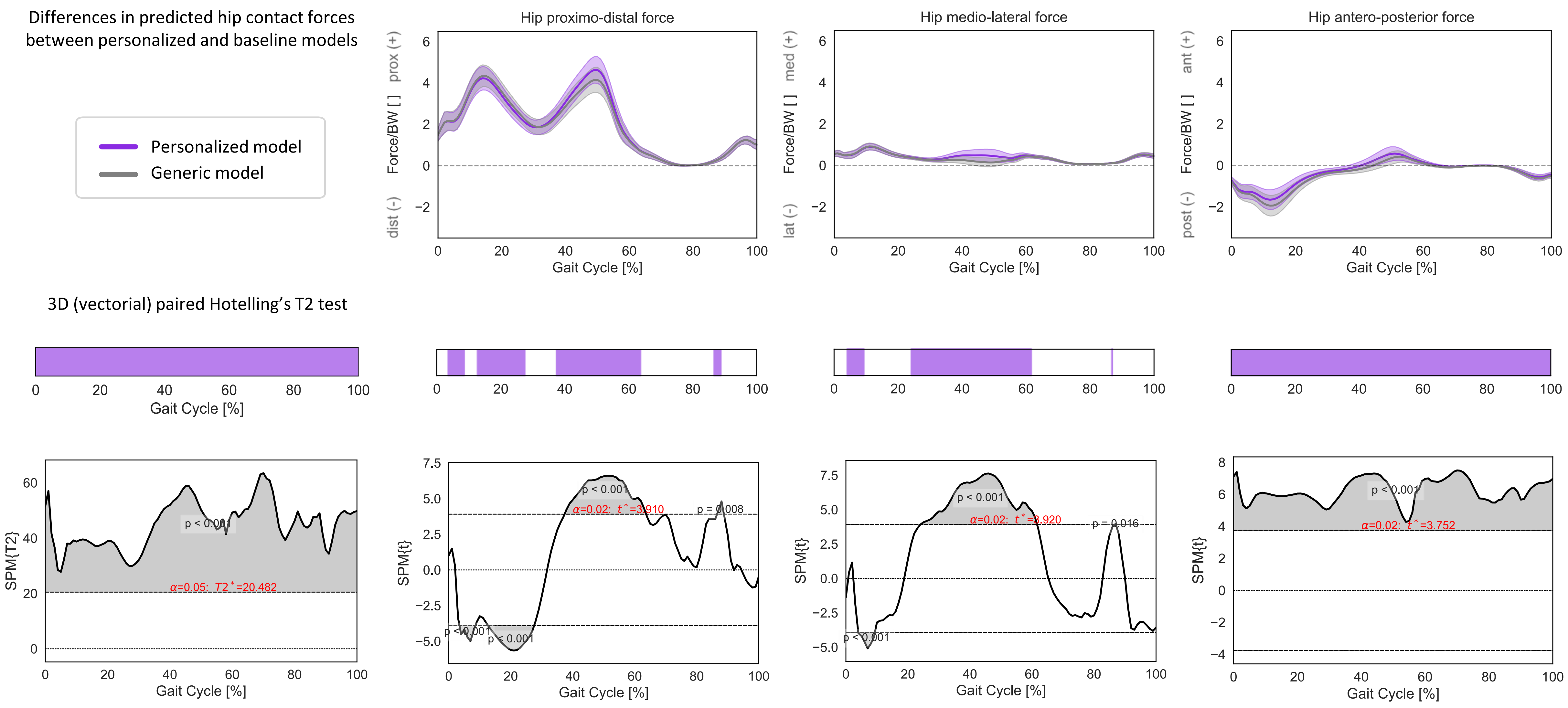

Differences in predicted hip contact forces between personalized and baseline models
Personalized model
Generic model
Hip proximo-distal force
Hip medio-lateral force
Hip antero-posterior force
Force/BW [ ]
prox (+)
dist (-)
med (+)
lat (-)
ant (+)
post (-)
Gait Cycle [%]
3D (vectorial) paired Hotelling's T2 test
SPM{T2}
SPM{t}
p < 0.001
α=0.05: T2*=20.482
α=0.02: t*=3.910
p = 0.008
α=0.02: t*=3.920
p = 0.016
α=0.02: t*=3.752
