# Supplementary material for: Subject-Specific Modeling of Femoral Torsion Influences the Prediction of Hip Loading During Gait in Asymptomatic Adults
Source: Front Bioeng Biotechnol. 2021 Jul 21;9:679360. doi: 10.3389/fbioe.2021.679360 (PMC8334869; doi:10.3389/fbioe.2021.679360)
Supplement: Supplementary file 1 [file Data_Sheet_1.pdf]

Hip flexors

15° retrotorsion | 5.5° antetorsion (baseline) | 25° antetorsion | 45° antetorsion | 45° antetorsion with 20° hip internal rotation

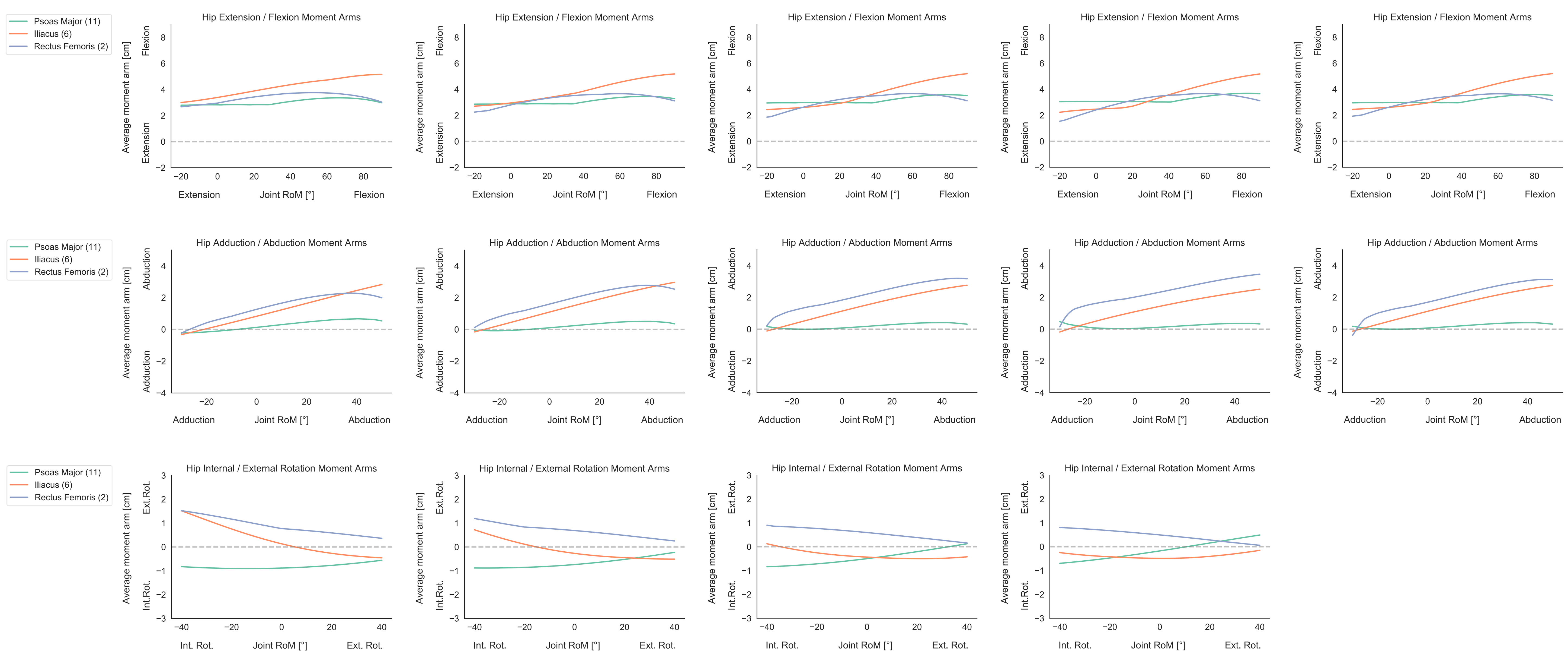

Hip extensors

15° retrotorsion | 5.5° antetorsion (baseline) | 25° antetorsion | 45° antetorsion | 45° antetorsion with 20° hip internal rotation

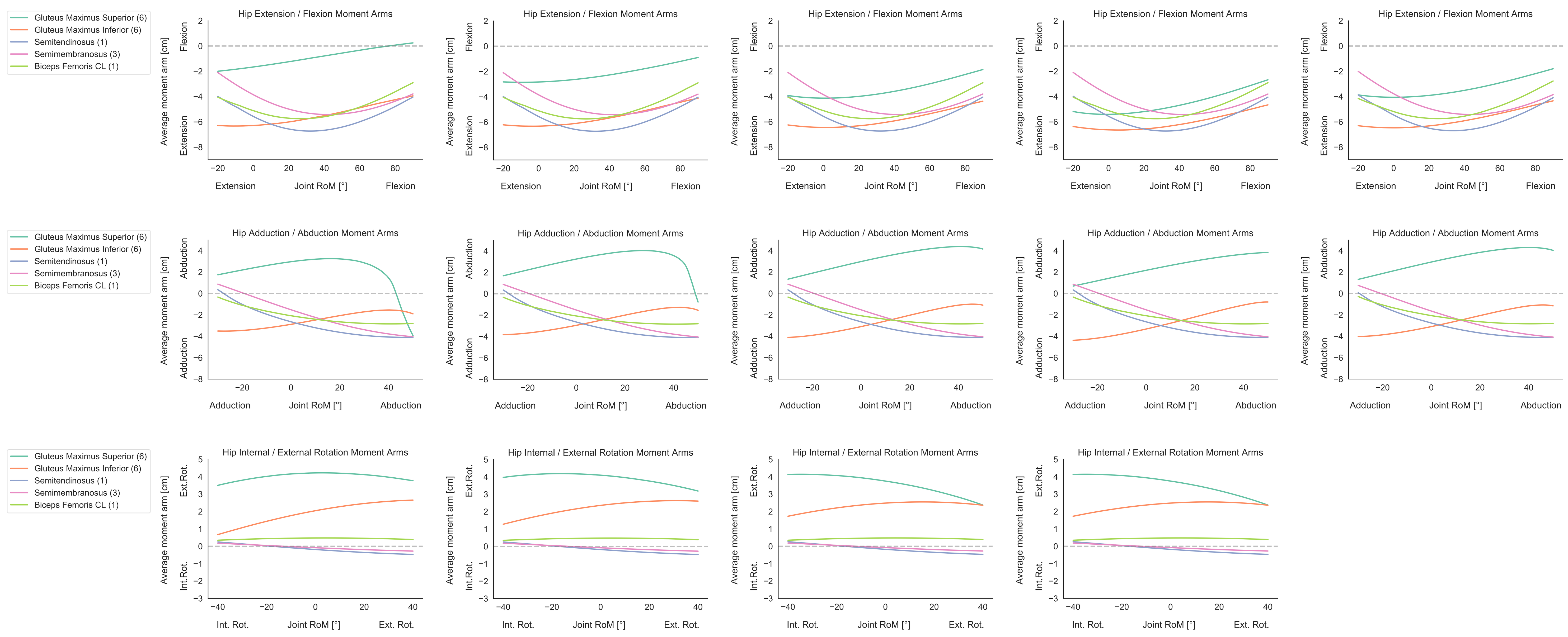

Hip abductors

15° retrotorsion | 5.5° antetorsion (baseline) | 25° antetorsion | 45° antetorsion | 45° antetorsion with 20° hip internal rotation

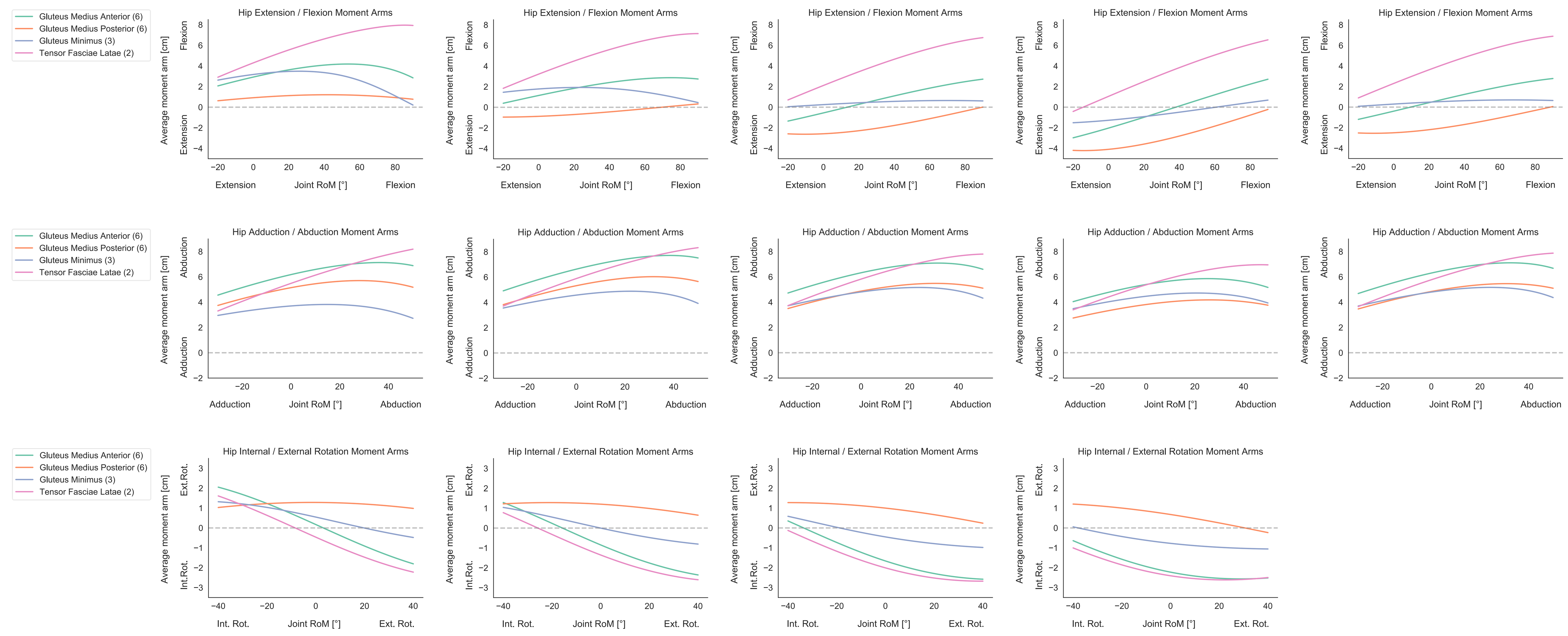

Hip adductors

15° retrotorsion | 5.5° antetorsion (baseline) | 25° antetorsion | 45° antetorsion | 45° antetorsion with 20° hip internal rotation

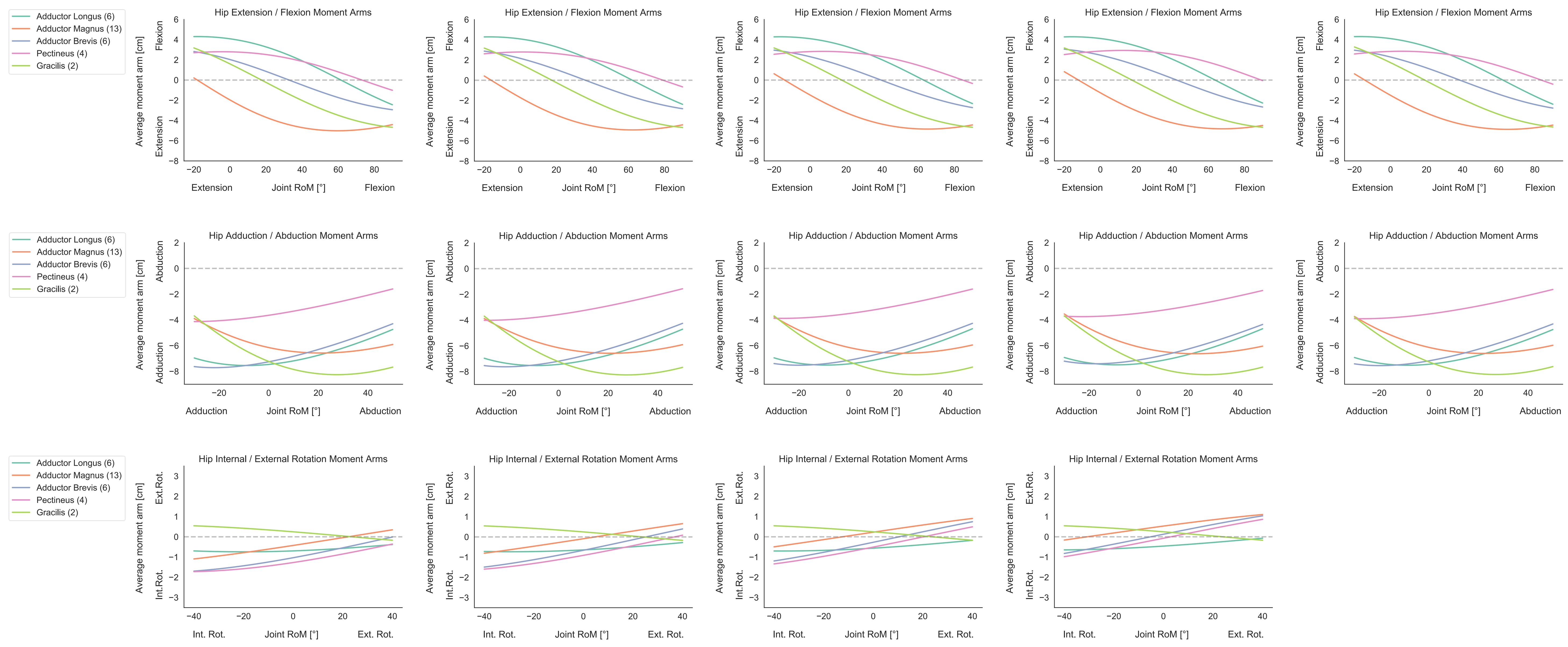

Muscle moment arms for different femoral morphologies:

Lever arms were calculated for each individual muscle fascicle across ranges of hip sagittal motion (20° extension, 90° flexion), hip frontal motion (30° adduction, 50° abduction), and hip transversal motion (40° internal, 40° external rotation), reported as rows. Average lever arms were calculated for the fascicles constituting each muscle and the specific number of fascicles is reported in brackets next to the muscle's name.

Four different modelled femoral morphologies were compared: the baseline unmorphed model, characterized by a femoral antetorsion of 5.5°, as well as morphed femurs with retrotorsion of -15° and antetorsion of +25° and +45°, reported as columns. Additionally, muscle lever arms were also calculated for the morphed femur with +45° of antetorsion and a fixed hip internal rotation angle of 20° (rightmost column), in order to mimic a compensatory kinematic strategy suspected to restore abduction capacity in pathological patients with torsional deformities.
